# Partial deficiency of HIF-1α stimulates pathological cardiac changes in streptozotocin-induced diabetic mice

**DOI:** 10.1186/1472-6823-14-11

**Published:** 2014-02-06

**Authors:** Romana Bohuslavova, Frantisek Kolar, David Sedmera, Lada Skvorova, Frantisek Papousek, Jan Neckar, Gabriela Pavlinkova

**Affiliations:** 1 Institute of Biotechnology AS CR, Prague, Czechia; 2 Institute of Physiology AS CR, Prague, Czechia; 3 Institute of Anatomy, First Faculty of Medicine, Charles University, Prague, Czechia; 4 Laboratory of Molecular Pathogenetics, Institute of Biotechnology AS CR, v.v.i., Videnska 1083, Prague 4, CZ-142 20, Czechia

**Keywords:** Echocardiographic parameters, Hypoxia inducible factor 1α, Diabetic cardiomyopathy, Vascular endothelial growth factor A, Heterozygous *Hif1a* knock-out

## Abstract

**Background:**

Diabetic cardiomyopathy is associated with a number of functional and structural pathological changes such as left ventricular dysfunction, cardiac remodeling, and apoptosis. The primary cause of diabetic cardiomyopathy is hyperglycemia, the metabolic hallmark of diabetes. Recent studies have shown that a diabetic environment suppresses hypoxia-inducible factor (HIF)-1α protein stability and function. The aim of this study was to analyze the functional role of HIF-1α in the development of diabetic cardiomyopathy. We have hypothesized that the partial deficiency of HIF-1α may compromise cardiac responses under diabetic conditions and increase susceptibility to diabetic cardiomyopathy.

**Methods:**

Diabetes was induced by streptozotocin in wild type (*Wt*) and heterozygous *Hif1a* knock-out (*Hif1a*^*+/-*^) mice. Echocardiographic evaluations of left ventricular functional parameters, expression analyses by qPCR and Western blot, and cardiac histopathology assessments were performed in age-matched groups, diabetic, and non-diabetic *Wt* and *Hif1a*^*+/-*^ mice.

**Results:**

Five weeks after diabetes was established, a significant decrease in left ventricle fractional shortening was detected in diabetic *Hif1a*^*+/-*^ but not in diabetic *Wt* mice. The combination effects of the partial deficiency of *Hif1a* and diabetes affected the gene expression profile of the heart, including reduced vascular endothelial growth factor A (*Vegfa*) expression. Adverse cardiac remodeling in the diabetic *Hif1a*^*+/-*^ heart was shown by molecular changes in the expression of structural molecules and components of the extracellular matrix.

**Conclusions:**

We have shown a correlation between heterozygosity for *Hif1α* and adverse functional, molecular, and cellular changes associated with diabetic cardiomyopathy. Our results provide evidence that HIF-1α regulates early cardiac responses to diabetes, and that HIF-1α deregulation may influence the increased risk for diabetic cardiomyopathy.

## Background

Both type 1 and type 2 diabetes are characterized by an increased risk of cardiomyopathy and myocardial infarction. Diabetic cardiomyopathy is associated with a number of functional and structural pathological changes, including decreased diastolic compliance, systolic dysfunction, apoptosis, and cardiac remodeling [1]. Diabetes is also associated with the majority of risk factors for cardiac failure, such as hypertension, hyperlipidemia, obesity, thrombosis, autonomic neuropathy, endothelial dysfunction, and microvascular pathology [2-4]. Hyperglycemia triggers diabetic tissue damage, including cardiovascular and microvascular complications. Existing evidence suggests that hyperglycemia induces an altered metabolism, abnormal expression of genes, the overproduction of reactive oxygen species (ROS), and mitochondrial dysfunction, which are underlying mechanisms behind pathological changes in diabetes [3]. Hypoxia is one of the most important pathophysiological factors associated with diabetic complications.

Transcriptional responses to hypoxia are mediated by hypoxia inducible factor 1 (HIF-1). HIF-1 activates over 800 target genes that are involved in cell proliferation, angiogenesis, glycolytic energy metabolism, and apoptosis [5]. HIF-1 consists of two subunits, HIF-1α, the regulatory subunit, and constitutively expressed HIF-1β. Oxygen tension plays a key role in the regulation of HIF-1α expression, stabilization, and activation [5]. The bulk of this response can be further modulated by growth factor and cytokine dependent signaling pathways [6,7]. Furthermore, existing evidence indicates that mitochondrial ROS are sufficient enough to initiate the stabilization and activation of HIF-1α, and that treatment with antioxidants prevents HIF-1α protein stabilization [8,9]. Embryonic lethality due to cardiovascular defects resulting from the global deletion of HIF-1α illustrates the critical role of *Hif1a* in embryonic development [10]. *Hif1a*^*+/-*^ mutants normally survive past embryonic development; however, *Hif1a* heterozygotes demonstrate impaired responses when challenged with hypoxia after birth [11,12]. A partial deficiency of HIF-1α has been associated with a complete loss of cardioprotection against ischemia–reperfusion injury, including the impairment of functional recovery parameters, a lack of ROS generation, and increased apoptosis [13]. Cardiac myocyte-specific HIF-1α gene deletion causes reductions in contractility, vascularization, and alters the expression of multiple genes in the heart during normoxia [14]. These findings point toward the central role of HIF-1α in coordinating molecular, cellular, and functional responses in the heart and, also, toward the central role of HIF-1α in diseases with impaired oxygen delivery, such as diabetes.

The diabetic environment reduces HIF-1α expression and function [15-18]. Consistent with a negative effect of diabetes on HIF-1α function, decreased levels of one of the best-known HIF-1 targets, VEGF-A, have been detected in diabetic hearts and other tissues [19]. In fact, the down-regulation of VEGF-A in diabetic hearts is the earliest event detected during diabetic cardiomyopathy and it is associated with the initiation of all the other features of diabetic cardiomyopathy, such as apoptosis, fibrosis, and progressive diastolic and systolic dysfunction [20]. Dysfunction of the left ventricle (LV) in diabetic cardiomyopathy has been correlated with cardiac remodeling, which leads to myocardial collagen deposition and cardiac fibrosis [21]. The causal relationship between decreased VEGFA expression, HIF-1α functional activity, and high glucose-induced microvascular pathology has been revealed in experiments with a wound healing mouse model [18]. Interestingly, an impaired ability to increase hypoxia-stimulated VEGF-A expression in diabetic tissues resulted from a primary defect in HIF-1 transactivation but not HIF-1 stabilization. HIF-1 activity increased by a local adenovirus-mediated transfer of stable HIF-1α constructs normalizes VEGF-A expression and prevents diabetic complications [15,16]. Cardiac-specific HIF-1α–overexpressing transgenic mice show cardiac protection from diabetes-induced defects in glucose metabolism and angiogenesis [22]. HIF-1α overexpression has restored VEGF-A levels and blocked cardiac fibrosis in the diabetic heart. However, the functional parameters of the LV have not been evaluated, which would be necessary for a more complex analysis.

Our study examines the relationship between the development of diabetic cardiomyopathy and the partial deficiency of HIF1-α caused by the global deletion of *Hif1a* functional allele. We have hypothesized that the partial deficiency of *Hif1a* may compromise cardiac responses under diabetic conditions and increase susceptibility to diabetic cardiomyopathy. Our research provides a new insight into the potential role of HIF-1α and *Hif1a* genetic variations in multiple pathways in diabetic cardiomyopathy. We analyzed echocardiographic parameters and molecular changes in the early phase of diabetic cardiomyopathy. We evaluated the expression of six HIF-1 transcriptional targets in order to identify signaling pathways and genes that may contribute to cardiac changes accompanying diabetes-induced cardiomyopathy and to directly evaluate HIF1-pathway responses. These genes encode molecules involved in vasculogenesis, glucose metabolism, insulin signaling, and autophagy (*Vegfa*, *Flt1*, *Slc2a1*, *Ldha*, *Igf2*, and *Bnip3l*). Additionally, we evaluated the expression of structural molecules (*Cxadr, Cx43*) and molecules associated with cardiac remodeling (*Col1, Pdgfra, Ctss, Tgfbr1, Itgav,* and *Il6st*). Our data showed that the partial deficiency of the *Hif1a* gene accelerated the progression of pathological changes induced by the diabetic environment in the heart, including significant changes in cardiac mechanical function and in cardiac gene expression.

## Methods

### Experimental animals

This study was conducted in accordance with the Guide for the Care and Use of Laboratory Animals (NIH Publication No. 85-23, revised 1996). The experimental protocol was approved by the Animal Care and Use Committee of the Institute of Molecular Genetics, the Czech Academy of Sciences. The experimental mice were housed in a controlled environment (23°C; 12-h light/dark cycle) with free access to water and a standard chow diet. All experiments were performed with male and female littermate mice that were either wild-type, *Hif1a*^*+/+*^ (*Wt*) or heterozygous *Hif1a* knock-out (*Hif1a*^*+/-*^) on a FVB background (strain code 207, Charles River). The heterozygous *Hif1a* mutants have the *Hif1a*^*tm1jhu*^ mutant allele in which exon 2, encoding the bHLH domain of the *Hif1a* gene, has been replaced by an intragenic deletion with a neomycin resistance (*neo*^*R*^) gene [10]. The heterozygous *Hif1a* deficient mice showed a partial loss of HIF-1α protein expression levels [23,24]. Offspring of *Wt x Hif1a*^*+/–*^ matings were genotyped by PCR, using genomic DNA isolated from tails and amplifying neomycin (*Neo*) and *Hif1a* exon 2 sequences [10,25]. Both *Neo* (463-bp) and *Hif1a* (317-bp) sequences were amplified from the DNA of *Hif1a*^+/-^ mice, whereas only *Hif1a* sequences were amplified from the DNA of *Wt* mice (*Hif1a*^+/+^), respectively. The sequences of the *Neo* primers were 5′-ACTGGCTGCTATTGGGCGAAGTG-3′ and 5′-GTAAAGCACGAGGAAGCGGTCAG-3′. The conditions for PCR were 94°C for 30 s, 48°C for 30 s, and 72°C for 30 s, for 40 cycles. The sequences of *Hif1a* exon 2 primers were 5′-TGTAGTCTCCTGCTAAAAG-3′ and 5′-TTATTCGAGTTAAGACAAAC-3′. The conditions for PCR were 94°C for 30 s, 63°C for 30 s, and 72°C for 30 s, for 40 cycles.

Diabetes was induced in mice 8-10 weeks of age by 2 intraperitoneal injections of 100 mg/kg body weight of streptozotocin (STZ; Sigma, St. Louis, MO), as described in [26]. The fasting blood glucose levels were measured by glucometer (COUNTOUR TS, Bayer, Switzerland) one week after the last STZ injection. Mice whose blood glucose levels exceeded 13.9 mmol/L were considered diabetic. The mice were analyzed after being diabetic for 5 weeks. The fasting blood glucose levels (mean ± SD) of *Wt* and *Hif1a*^*+/-*^ mice were 9.9 ± 0.3 and 10.0 ± 0.3 mmol/L before STZ treatment, and 32.5 ± 1.8 and 30.3 ± 2.0 mmol/L after 5 weeks of diabetes, respectively.

### Echocardiography

The echocardiographic evaluation of the geometrical and functional parameters of the LV was performed using the GE Vivid 7 Dimension (GE Vingmed Ultrasound, Horten, Norway) with a 12 MHz linear matrix probe M12L. The animals were anesthetized by the inhalation of 2% isoflurane (Aerrane, Baxter SA) and their rectal temperature was maintained within 36.5 and 37.5°C by a heated table throughout the measurements. For the baseline evaluation, the following diastolic and systolic dimensions of the LV were measured: the posterior wall thickness (PWT_D_ and PWT_S_), anterior wall thickness (AWT_D_ and AWT_S_), and the cavity diameter (LVD_D_ and LVD_S_). From these dimensions, the main functional parameter, fractional shortening (FS) was derived by the following formula: FS [%] = 100 × (LVD_D_ – LVD_S_)/LVD_D_.

After the echocardiographic examination, a fluid filled catheter connected to an external transducer (Bpr-02, Experimetria) was introduced into the left carotid artery to measure the blood pressure. The mean blood pressure was averaged from five measurements within a 10-min interval. The hearts were then rapidly excised and dissected into the right ventricle (RV), the LV and the interventricular septum. All ventricular parts were weighed and processed for subsequent analyses.

### Quantitative real-time PCR

RNA was isolated from the LV of individual diabetic and non-diabetic adult males (8 individual samples/each group) by Trizol® (Invitrogen). The concentration of extracted RNA was quantified using NanoDrop. Quantitative Real-Time PCR (RT-qPCR) was performed using the LightCycler® 480 Real-Time PCR system (Roche, Roche Applied Science, Mannheim, Germany) on cDNA samples. The collected RNA samples (1 μg) were subjected to reverse transcription using Superscript II (Fermentas, Lithuania). cDNA was diluted 20× and 4 μl were added to 6 μl of Syber® Green JumpStart™ Tag ReadyMix™ (Sigma) with primers (0.25 μmol). Following the reverse transcription (RT), quantitative real-time PCR (qPCR) was performed with the initial AmpliTaq activation at 95°C for 10 min, followed by 40 cycles at 95°C for 15 s and 60°C for 60 s, as described in [25]. The *Hprt1* gene was selected as the best reference gene for our analyses from a panel of 12 control genes (TATAA Biocenter AB, Sweden). The expression of this reference gene was unchanged in response to the experimental conditions being investigated. The relative expression of the target gene was calculated using the ΔΔCp method, based on qPCR efficiencies (E) and the crossing point (Cp) difference (Δ) of an experimental sample (diabetic *Wt*, non-diabetic and diabetic *Hif1a*^*+/-*^) versus control-non-diabetic *Wt* (ratio = (*E*_target_)^ΔCp target(Mean control – Mean EXP)/^(*E*_Hprt1_)^ΔCp Hprt1(Mean control – Mean EXP)^[27]. RT-qPCR data were analyzed using the GenEX5 program (http://www.multid.se/genex). The differences in normalized Cp values were tested for statistical significance. The primers were designed using the Primer 3 software (http://bioinfo.ut.ee/primer3/). Primer sequences are listed in Additional file 1: Table S1.

### Morphological analysis

The adult hearts of diabetic and non-diabetic *Wt* and *Hif1a*^*+/-*^ males were arrested in diastole by coronary perfusion with saline containing 5 mM cadmium chloride and 20 mM potassium chloride. After fixation with 4% paraformaldehyde overnight, the hearts were processed for paraffin histology. Adjacent sections (7 μm) were stained with Alcian Blue/Hematoxylin-Eosin (general histological staining), Picrosirius Red (PSR, collagen), TUNEL (apoptosis; #1684795, Roche), anti-collagen 1 (anti-Col1; #203002, MD Biosciences), anti-smooth muscle actin (#A 2574, Sigma), anti-CD34 (blood vessels; #ab8158, Abcam), anti-VEGF-A (#sc-7269, Santa Cruz Biotechnology), and anti-connexin43/wheat germ agglutinin (anti-Cx43/WGA; gap junctions and cell membranes; #C6219, Sigma/#W7024, Invitrogen). The nuclei were counterstained with Hoechst 33342 in fluorescence techniques or hematoxylin in diaminobenzidine (DAB; #D3939, Sigma) visualization protocol. Myocyte size (minimum transverse diameter) was measured on sections stained with anti-CD34 visualized by DAB. The cardiomyocytes can be best approximated as rod-shape with an oval cross section. Any errors due to a variation of the section plane are avoided by choosing the minor axis only in cells where a nucleus is present. Each analysis was repeated a minimum of 2 times on 2-3 individual samples per genotype and included appropriate controls. The sections were analyzed under a Nikon Eclipse E400 fluorescent microscope or Leica SPE confocal microscope with a 40× magnification oil immersion objective, with NIS-elements or LCS program. VEGF-A^+^ areas were quantified using Image J software. The evaluator of the VEGF-A expression was blinded to the experimental conditions and genotype.

### Western blot

Dissected LVs from the diabetic and non-diabetic hearts were lysed with protease and phosphatase inhibitors to prevent protein degradation and stored at –80°C until analysis. For HIF1-α immunoblot assays, nuclear extracts from dissected LVs were prepared using a Nuclear extract kit (#40010; Active Motif, Belgium). The protein levels were quantified using the BCA assay. 20 μg of total protein lysates or 30 μg of nuclear extracts per lane were denatured, resolved using 10% SDS-PAGE, and transferred to a nitrocellulose membrane [10,28]. The membrane was blocked with 5% dry milk and incubated overnight with rabbit anti-Col1 at 1: 1000 dilution (#203002; MD bioproducts, Switzerland), anti-Cx43 at 1:6000 (#C6219, Sigma), anti-HIF-1α at 1:750 dilution (#NB100-105; Novus 224 Biologicals, UK), or anti-phospho-Cx43 at 1:1000 (#3511; Cell Signaling, MA, USA). After incubation with a horseradish peroxidase–conjugated secondary IgG (Amersham, IL, USA), the blots were developed using the SuperSignal* West Dura Chemiluminescent Substrate (#PIA34075; Thermo Scientific, MI, USA). Chemiluminescent signals were captured using an ImageQuant LAS 4000 Imager (GE Healthcare Bio-Sciences AB, Sweden) and analyzed by ImageJ software (http://rsbweb.nih.gov/ij/). Protein levels were quantified on duplicate blots and were normalized to the loading control mitochondrial membrane marker (ATP5a) or glyceraldehyde 3-phosphate dehydrogenase (GAPDH; Membrane Fraction WB Cocktail, #ab140365; Abcam, Cambridge, USA).

### Statistical analysis

All values are expressed as mean ± SEM. Group data were analyzed using 2-way ANOVA (with genotype and experimental condition as categories) and Tukey’s post-hoc multiple-comparisons test for between-group differences (significance assigned at the *P* < 0.05 level; Graph Pad, 2005; Graph Pad, San Diego, CA). Sample sizes and individual statistical results for all analyses are provided in the figures and tables.

## Results

### Echocardiographic evaluation of the LV function

**Figure 1:**
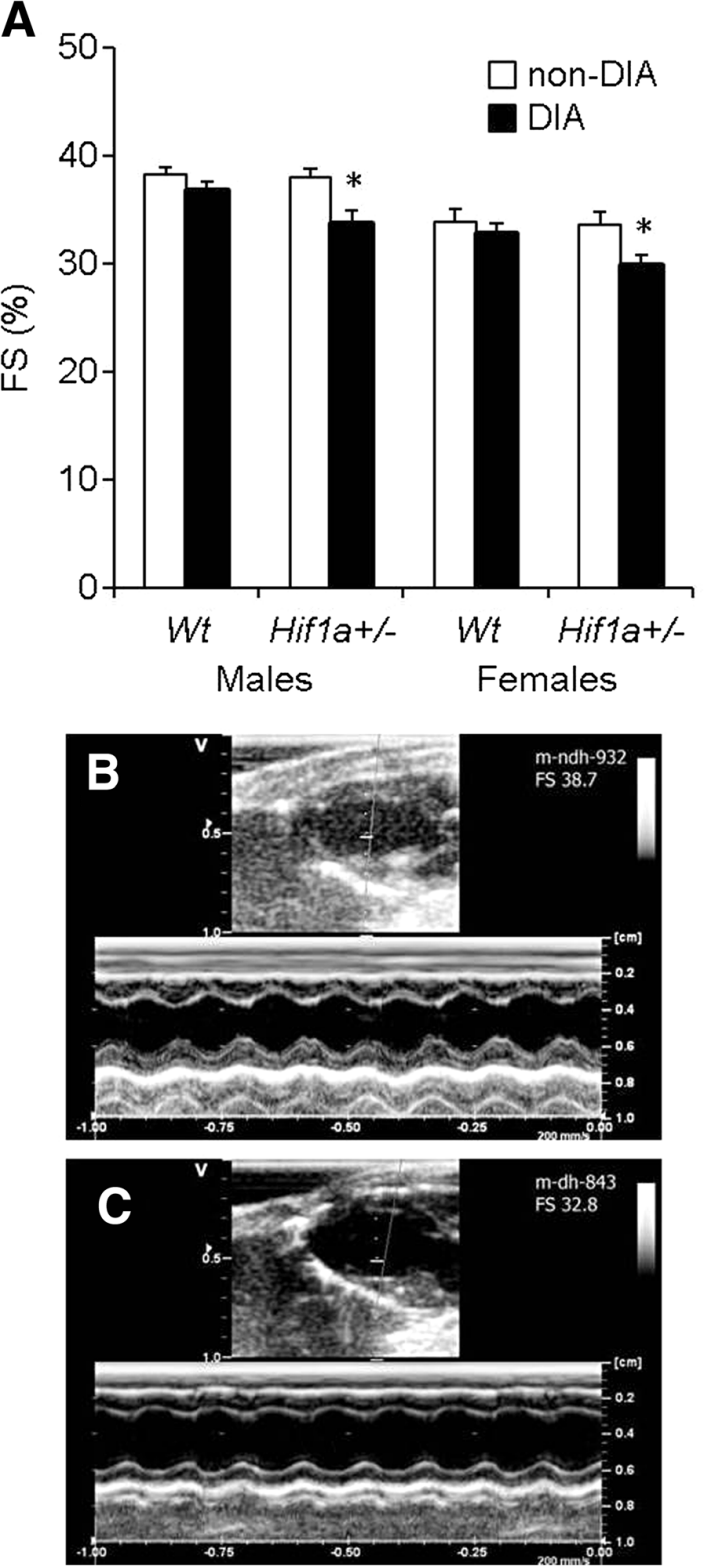
**Echocardiographic assessment of LV systolic function in *Wt *and *Hif1a***^***+/-***^**mice. (A)** The exposure to the diabetic environment resulted in a significant decrease of LV fractional shortening (FS) in *Hif1a*^*+/-*^ mice but not in *Wt* animals. The values are mean ± SEM, statistical significance by 2-way ANOVA with Tukey’s multiple-comparison test, *P < 0.05, diabetic *Hif1a*^*+/-*^*vs. Wt* and *Hif1a*^*+/-*^ non-diabetics. Representative M-mode recordings of LV structures in long axes view in a non-diabetic **(B)** and diabetic **(C)***Hif1a*^*+/-*^ mouse with FS = 38.7 and FS = 32.8, respectively.

Five weeks after diabetes was induced by repeated intraperitoneal STZ injections, the body mass and LV mass gains of diabetic *Hif1a*^*+/-*^ and *Wt* males were lower compared to non-diabetic groups (Table 1). Diabetic females were less affected in the body and LV mass gains than diabetic males. Neither heart rate nor blood pressure differed among the groups, although blood pressure tended to increase in both *Wt* and *Hif1a*^*+/-*^ diabetic mice compared to the corresponding controls (males: *P* = 0.053 and 0.066, respectively, Table 2). LV echocardiography did not reveal any difference between non-diabetic *Wt* and *Hif1a*^*+/-*^ mice. However, diabetes significantly influenced the LV echocardiographic parameters of *Hif1a*^*+/-*^ mice. The general trends of functional changes induced by diabetes were similar in males and females (Figure 1 and Table 2). LV FS was unaffected by genotype in non-diabetic *Wt* (males: 38.3 ± 0.6, females: 33.8 ± 1.3) and non-diabetic *Hif1a*^*+/-*^ mice (males: 38.0 ± 0.9, females: 33.6 ± 0.6, Figure 1A). A significant decrease in LV FS was detected in diabetic *Hif1a*^*+/-*^ mice (males: 33.8 ± 1.2, females: 29.8 ± 1.0) but not in diabetic *Wt* animals (males: 36.9 ± 0.8, females: 32.8 ± 0.9, Figure 1A). The differences in LV FS between non-diabetic and diabetic *Hif1a*^*+/-*^ mice are also shown in representative M-mode echocardiographic recording (Figure 1B and C). Both diastolic and systolic AWT and PWT were significantly lower in diabetic *Hif1a*^*+/-*^ males than in non-diabetic *Wt* and *Hif1a*^*+/-*^ males. Although we observed similar tendencies in AWT and PWT parameters in diabetic *Wt* animals, these differences were not statistically significant. Thus, the partial deficiency of *Hif1a* compromised the LV functional parameters under diabetic conditions.

**Table 1:** Body and heart mass

**Genotype**	***Wt***	***Wt***	***Hif1a***^***+/-***^	***Hif1a***^***+/-***^
**Parameter**	**Non-diabetic**	**Diabetic**	**Non-diabetic**	**Diabetic**
Males (n)	7	8	10	8
BM (g)	28.0 ± 0.4	24.8 ± 0.7*	28.4 ± 0.6	22.9 ± 0.8*^†^
HM (mg)	106 ± 2	88 ± 3*	104 ± 4	86 ± 3*^†^
RV/BM	0.77 ± 0.03	0.74 ± 0.04	0.76 ± 0.04	0.73 ± 0.02
LV/BM	2.18 ± 0.03	1.98 ± 0.06*	2.07 ± 0.04	1.96 ± 0.03*
Females (n)	7	12	10	8
BM (g)	22.0 ± 0.7	22.1 ± 0.6	22.1 ± 0.5	20.5 ± 0.4
HM (mg)	81 ± 1	80 ± 1	79 ± 2	77 ± 1
RV/BM	0.80 ± 0.04	0.80 ± 0.02	0.74 ± 0.02	0.73 ± 0.03
LV/BM	2.34 ± 0.07	2.31 ± 0.08	2.26 ± 0.05	2.34 ± 0.14

Values are means ± SEM from indicated number of animals in each group. Abbreviations: BM, body mass; HM, heart mass; LV, left ventricular mass; RV, right ventricular mass. Statistical significance was assessed by 2-way ANOVA with Tukey’s multiple-comparison test, *P < 0.05 *vs*. non-diabetic *Wt*; ^†^*P* < 0.05 *vs.* non-diabetic *Hif1a*^*+/-*^.

**Table 2:** Mean systemic arterial blood pressure (MAP), heart rate (HR) and basal left ventricular echocardiographic parameters

**Genotype**	***Wt***	***Wt***	***Hif1a***^**+/-**^	***Hif1a***^**+/-**^
**Parameter**	**Non-diabetic**	**Diabetic**	**Non-diabetic**	**Diabetic**
Males (n)	6	8	10	8
MAP (mmHg)	83.0 ± 2.1	87.5 ± 1.5	84.1 ± 1.4	88.2 ± 1.6
HR (beats/min)	496 ± 23	471 ± 13	449 ± 40	440 ± 36
LVD_D_ (mm)	3.60 ± 0.09	3.59 ± 0.04	3.47 ± 0.10	3.65 ± 0.06
LVD_S_ (mm)	2.22 ± 0.07	2.27 ± 0.05	2.16 ± 0.08	2.42 ± 0.07
AWT_D_ (mm)	0.84 ± 0.02	0.76 ± 0.01	0.89 ± 0.02	0.74 ± 0.03^†^*
PWT_D_ (mm)	0.84 ± 0.03	0.76 ± 0.01	0.92 ± 0.04	0.69 ± 0.03^†^*
AWT_S_ (mm)	1.30 ± 0.03	1.20 ± 0.04	1.27 ± 0.03	1.10 ± 0.05^†^*
PWT_S_ (mm)	1.21 ± 0.03	1.09 ± 0.03	1.20 ± 0.04	1.00 ± 0.04^†^*
Females (n)	7	12	10	8
MAP (mmHg)	84.6 ± 2.1	88.5 ± 2.2	84.8 ± 0.04	88.1 ± 1.3
HR (beats/min)	504 ± 7	478 ± 15	484 ± 16	449 ± 17
LVD_D_ (mm)	3.52 ± 0.08	3.52 ± 0.06	3.45 ± 0.04	3.46 ± 0.04
LVD_S_ (mm)	2.33 ± 0.07	2.37 ± 0.06	2.30 ± 0.05	2.43 ± 0.06
AWT_D_ (mm)	0.75 ± 0.02	0.75 ± 0.01	0.72 ± 0.02	0.72 ± 0.02
PWT_D_ (mm)	0.79 ± 0.02	0.74 ± 0.02	0.78 ± 0.03	0.74 ± 0.02
AWT_S_ (mm)	1.12 ± 0.04	1.05 ± 0.02	1.06 ± 0.01	0.98 ± 0.02^†^*
PWT_S_ (mm)	1.10 ± 0.02	1.05 ± 0.02	1.09 ± 0.02	0.99 ± 0.02*

Values are mean ± SEM. LVD_D_ - diastolic cavity diameter, LVD_S_ - systolic cavity diameter, AWT_D_ - diastolic anterior wall thickness, PWT_D_ - diastolic posterior wall thickness, AWT_S_ - systolic anterior wall thickness, PWT_S_ - systolic posterior wall thickness. Statistical significance was assessed by 2-way ANOVA with Tukey’s multiple-comparison test, *P < 0.05 *vs*. non-diabetic *Wt*, ^†^*P* < 0.05 *vs*. non-diabetic *Hif1a*^*+/-*^.

Since male groups were more affected in LV echocardiographic parameters by diabetes than female groups, we only used males for our subsequent analyses.

### Cardiac gene expression profiling

**Figure 2:**
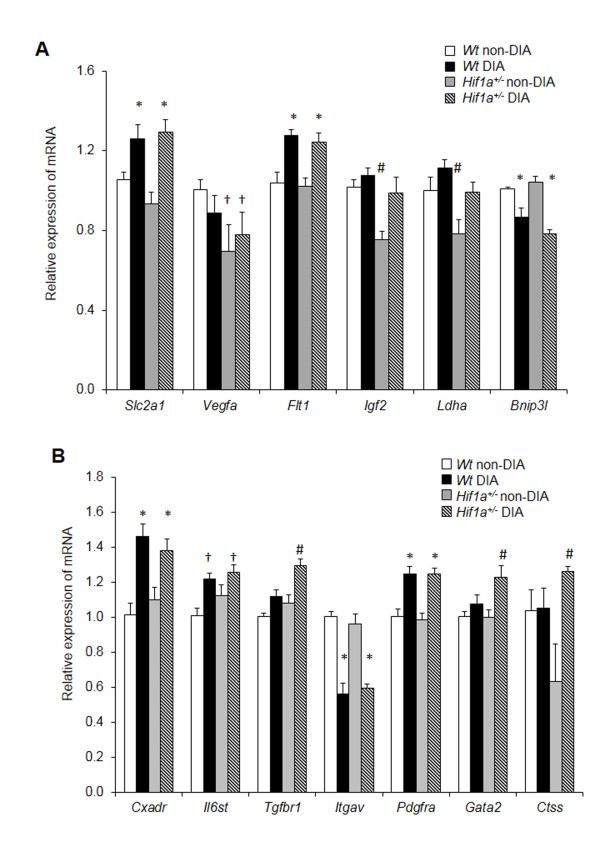
**Gene expression changes in the LV of *Wt *and *Hif1a***^***+/-***^**diabetic mice.** The expression of genes was analyzed using RT-qPCR: **(A)** direct HIF-1α target genes and **(B)** genes encoding signaling molecules, growth factors, cytokines, and transcription factors. The relative expression levels were quantified using the ΔΔCT method. The data represent the expression of mRNA relative to the non-diabetic *Wt* expression of mRNA, normalized by the housekeeping mRNA of *Hprt1*. The values are mean ± SEM (each experiment in duplicate; n = 8). All tests showed a significant effect of diabetes in a 2-way ANOVA, *P* < 0.01. The effect of genotype was significant in a 2-way ANOVA for *Igf2* (*P* < 0.003), *Ldha* (*P* < 0.0001), *Tgfbr* (*P* < 0.006), *Gata2* (*P* < 0.01), *Ctss* (*P* < 0.05). We identified a condition-genotype interaction (2-way ANOVA interaction effect *P* < 0.01) for *Vegfa*. Tukey’s post-hoc multiple-comparison test was used for between-group differences, *P < 0.05 *vs. Wt* and *Hif1a*^*+/-*^ non-diabetics, ^†^*P* < 0.05 *vs.* non-diabetic *Wt*, ^#^*P* < 0.05 *vs.* all other groups. Abbreviations: glucose transporter 1 (*Slc2a1*), vascular endothelial growth factor A (*Vegfa*), Vegf receptor-1 (*Flt1*), insulin-like growth factor 2 (*Igf2*), lactate dehydrogenase A (*Ldha*), BCL2/adenovirus E1B interacting protein 3-like (*Bnip3l*), coxsackie virus and adenovirus receptor (*Cxadr*), interleukin 6 signal transducer (*Il6st*), transforming growth factor beta receptor I (*Tgfbr1*), integrin alpha V (*Itgav*), platelet derived growth factor receptor alpha (*Pdgfra*), GATA binding protein 2 (*Gata2*), cathepsin S (*Ctss*).

To explore the tissue specific molecular changes induced by diabetes, we analyzed the expression of 13 selected genes in the LV myocardium (Figure 2). All tests showed a significant effect of diabetes. We analyzed the expression of six HIF-1 target genes involved in glucose metabolism (*Ldha*; *Slc2a1*), autophagy (*Bnip3l*), insulin signaling (*Igf2*) and vasculogenesis (*Vegfa*; *Flt1*; Figure 2A). Under normal conditions, the HIF-1α heterozygous-null mutants showed a decreased cardiac transcription of three HIF-1 target genes, *Vegfa*, *Igf2*, and *Ldha*, reflecting *Hif1a* haploinsufficiency. The expression levels of mRNA of *Vegfa* were significantly affected by the combination of genotype and diabetes (2-way ANOVA interaction effect, *P* < 0.01). The cardiac expression of *Slc2a1, Flt1*, and *Bnip3l* mRNA was significantly affected by diabetic conditions, but not by genotype. We also analyzed the expression of additional genes encoding molecules associated with cardiac remodeling (Figure 2B). The expression levels of *Cxadr, Pdgfra,* and *Il6st* were increased, whereas the expression of *Itgav* was decreased in both *Wt* and *Hif1a*^*+/-*^ diabetic hearts compared to the non-diabetics. Interestingly, *Tgfbr1, Ctss,* and transcription factor *Gata2* levels were increased in the diabetic *Hif1a*^*+/-*^, but not in the diabetic *Wt* hearts (a significant effect of genotype, *P* < 0.01). Based on the gene expression analysis, we can conclude that the diabetic *Hif1a*^*+/-*^ hearts demonstrated molecular changes associated with transcriptional regulation and cardiac remodeling processes.

### Analysis of HIF-1α protein levels in the LV

**Figure 3:**
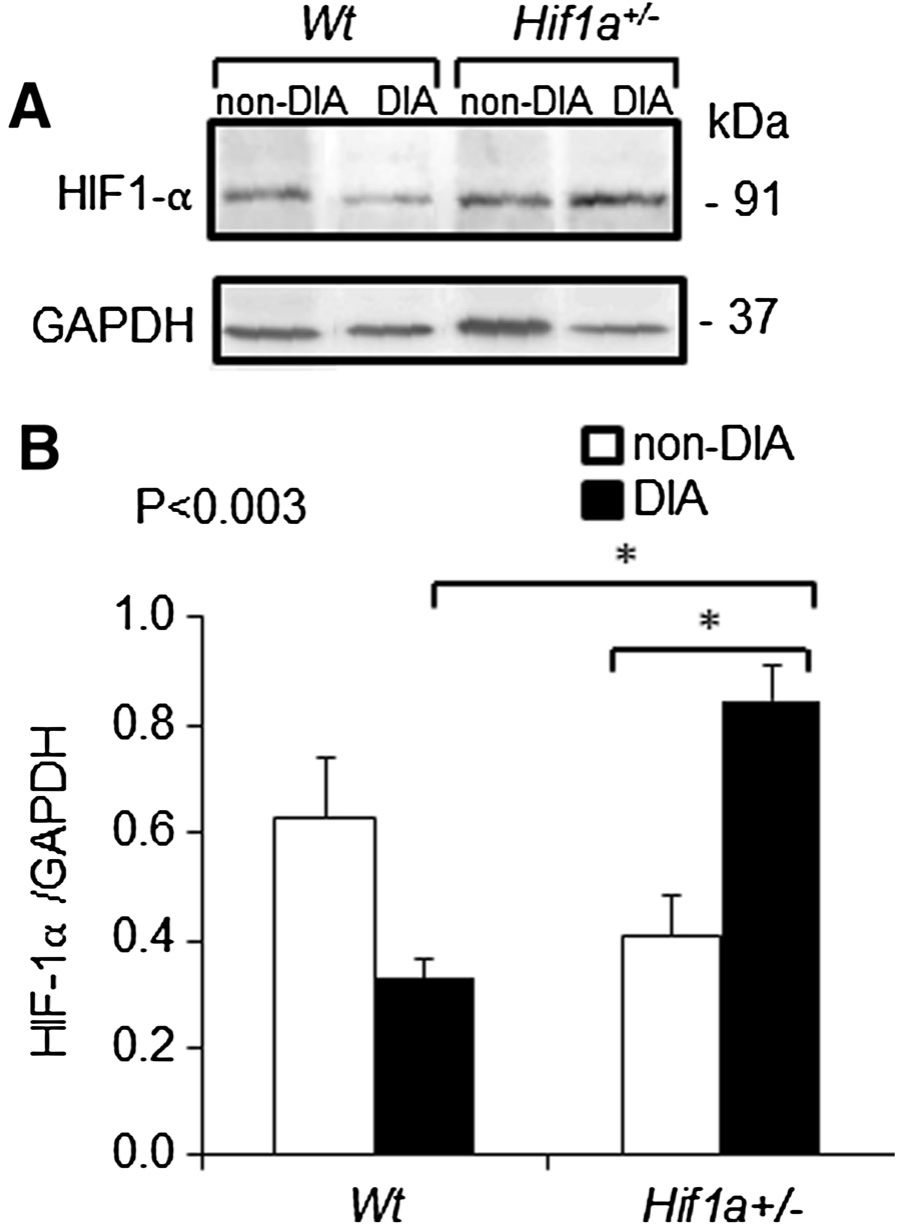
**Protein levels of HIF-1α in the LV of *Wt *and *Hif1a+/-*. (A)** The HIF-1α protein in nuclear fractions from the isolated LVs of *Wt* and *Hif1a+/-* non-diabetic and diabetic hearts was detected by Western blot analysis. A representative immunoblot is shown. **(B)** Bar graph shows mean ± SEM (n = 4/group) from densitometric analysis. The values are shown as a ratio of HIF-1α/loading control, GAPDH. *P* values shown are for the interaction of genotype and condition (2-way ANOVA), Tukey’s post-test, *P < 0.05.

In the next step, HIF-1α protein expression was analyzed in nuclear extracts from the LVs in order to understand the basis for the diabetes-induced changes in *Hif1a*^*+/-*^ diabetic hearts. A representative example of the immunoblot assay and the mean data obtained from densitometric analysis are presented in Figure 3A. Protein analysis revealed that HIF1-α levels were decreased by 35% in the LV of *Hif1a*^*+/-*^ hearts compared to *Wt*. A significant condition-genotype interaction was identified (*P* < 0.003, 2-way ANOVA). Unexpectedly, HIF-1α levels were significantly increased in diabetes-exposed *Hif1a*^*+/-*^ hearts compared to diabetes-exposed *Wt* and non-diabetic *Hif1a*^*+/-*^ mice by 2.6-fold and 2.1-fold, respectively. We detected a decreased HIF-1α expression in the diabetic *Wt* heart, although the difference was not statistically significant compared to non-diabetic *Wt* (*P* > 0.05, Figure 3B).

### Cellular and structural analysis

**Figure 4:**
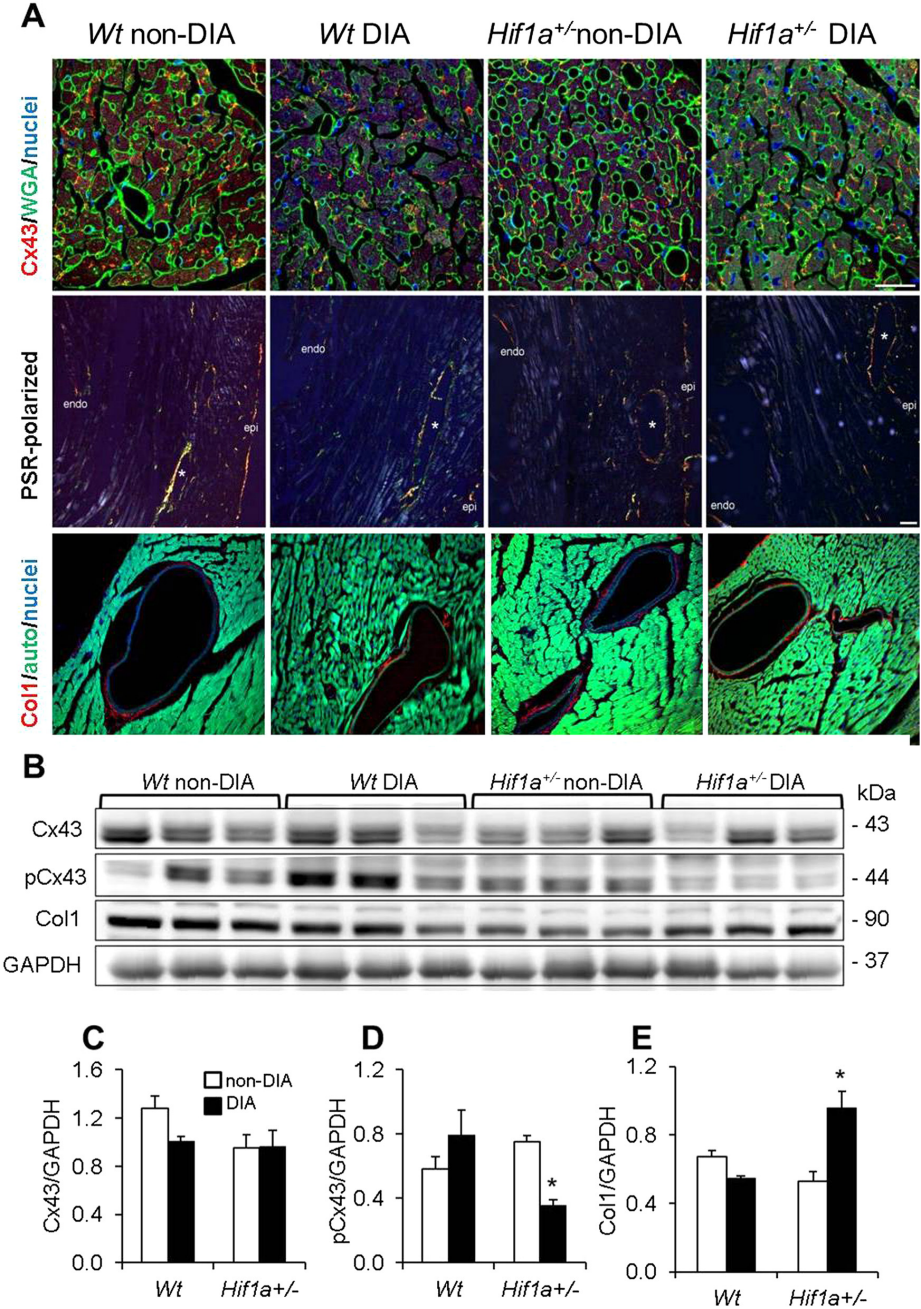
**Effects of diabetes on structural remodeling and protein levels in the *Wt *and *Hif1a***^***+/-***^**LV. (A)** Representative of immunofluorescence confocal and light microscopy images of the hearts stained with anti-connexin 43 (Cx43, red) together with fluorescein-labeled wheat germ agglutinin (WGA, green); with picrosirius red (PSR, polarizing microscopy); and with anti-collagen 1 (Col1, red), autofluorescence (auto) are shown. The nuclei were counterstained with Hoechst 33342 (blue). Confocal images are stacked Z-plane sections from confocal microscopy. Scale bar: Cx43/WGA 25 μm, PSR 50 μm, Col1 25 μm. **(B)** Representative Western blot analyses of protein lysates from the isolated LVs of *Wt* and *Hif1a*^*+/-*^ non-diabetic and diabetic hearts are shown. **(C-E)** A relative quantification of protein levels of Cx43, phosphoCx43 (pCx43), and Col1 (n = 3 per group) was performed. The bar graphs show the mean values of relative protein levels normalized to the loading control (GAPDH) ± SEM from densitometric analysis. *P < 0.05 (2-way ANOVA with Tukey’s post-test).

We further investigated pathogenic molecular and cellular changes associated with diabetes-induced myocardial remodeling, characterized by structural modifications, increased extracellular matrix and fibrosis, increased cardiac hypertrophy, apoptosis, and microvascular changes. The expression and proportion of phosphorylated and dephosphorylated forms of structural gap-junctional protein Cx43 are altered in diabetic conditions [29]. In our study, the relative abundance of Cx43 was moderately decreased in the diabetic myocardium compared to non-diabetic groups (Figure 4A). The quantification of Cx43 protein levels by Western blot showed no significant differences between groups or genotypes (Figure 4B and C). However, the phosphorylated form of Cx43 at serine 368 (Ph-Cx43) was significantly decreased in the diabetic *Hif1a*^*+/-*^ mutant LV (Figure 4D). Tissue sections from *Wt* and heterozygous *Hif1a*^*+/-*^ hearts were subjected to histological analysis to evaluate perivascular fibrosis by collagen deposition (PSR and anti-Col1 staining, Figure 4A). Perivascular collagen deposition was not noticeably different in diabetics compared to non-diabetic groups (Figure 4A). However, Western blot analysis detected a significant increase in the protein levels of Col1 in the LV of diabetic *Hif1a*^*+/-*^ heart compared to other analyzed groups (Figure 4E). Quantitative measurements of myocyte width yielded identical values in all groups (data not shown), which confirmed the absence of hypertrophy at this stage.

**Figure 5:**
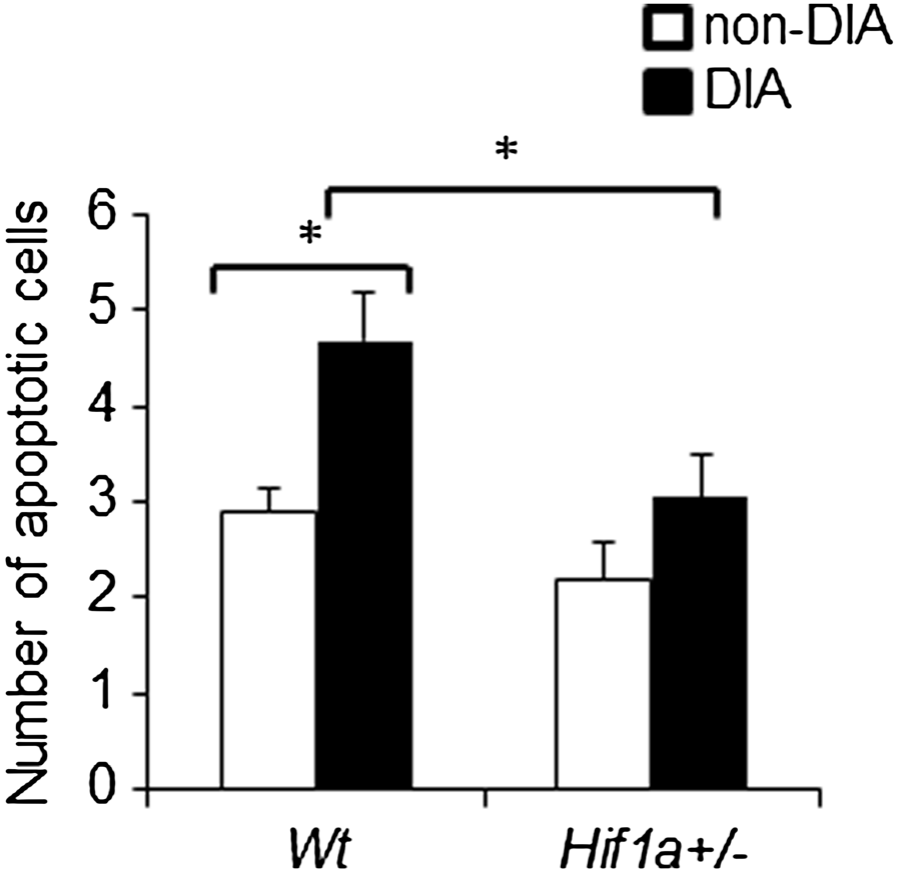
**Apoptosis in the diabetic and non-diabetic hearts of *Wt *and *Hif1a***^***+/-***^**mice.** The apoptotic cells were detected with TUNEL assay. The apoptotic cells were counted in the whole heart, including the myocardium of left and right ventricles, and the atrioventricular septum. The values represent mean ± SEM (n = 3 individuals × 3 heart sections per group); *P < 0.05, (2-way ANOVA with Tukey’s post-test).

Additionally, we analyzed levels of apoptosis using TUNEL staining. We counted apoptotic cells in the LV, RV, and septum. The number of apoptotic cells was moderately increased in the diabetic *Wt* but not in the diabetic *Hif1a*^*+/-*^ hearts, which suggests that the *Hif1a*^*+/-*^ genotype affects the apoptotic process in diabetic hearts (Figure 5).

**Figure 6:**
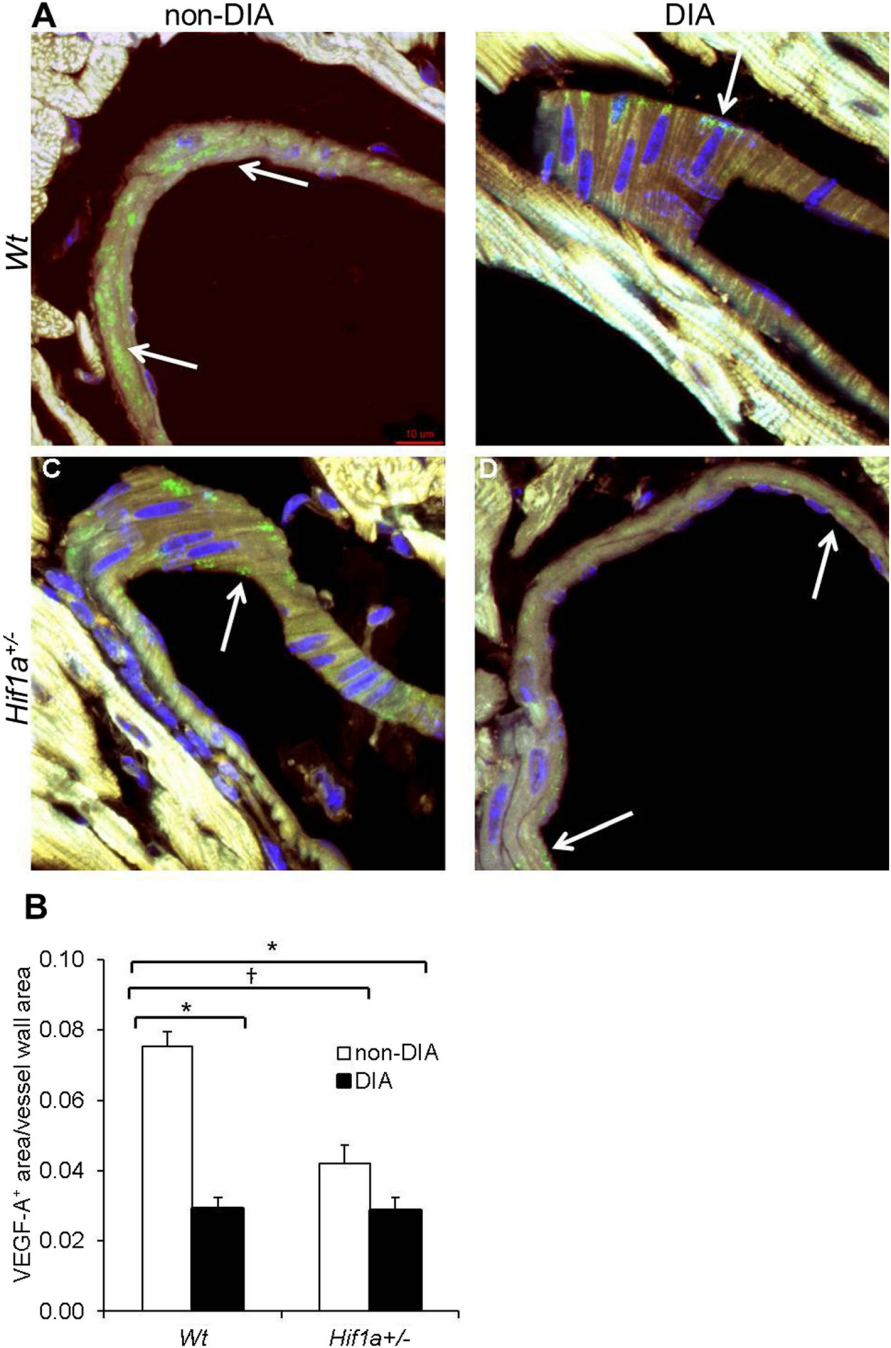
**Diabetes-induced changes in cardiac VEGF-A expression. (A)** Confocal imaging of transverse sections of *Wt* and *Hif1a*^*+/-*^ hearts stained with anti-VEGF-A antibody (green) showed VEGF-A expression in coronary blood vessels (white arrow). Hoechst 33342 (blue) was used as a nuclear counterstain. Images are stacked Z-plane sections from confocal microscopy. Scale bar: 10 μm. **(B)** A relative quantification of VEGF-A expression in the blood vessel wall was performed. The quantification of VEGF-A^+^ area was determined as a ratio of VEGF-A^+^ area per total vessel area in the field of view using ImageJ. Data are presented as the mean ± SEM (n = 4 - 8 vessels × 2 individuals per group); ^†^*P* < 0.01, non-diabetic *Wt* vs. non-diabetic *Hif1a*^*+/-*^; *P < 0.001, non-diabetic *Wt vs*. diabetic *Wt* or diabetic *Hif1a*^*+/-*^ (2-way ANOVA with Tukey’s post-test).

Since our RT-qPCR analysis demonstrated a significant combinatorial effect of genotype and diabetes on *Vegfa* mRNA expression, we analyzed the cardiac expression of VEGF-A, a key HIF-1 target gene product. VEGF-A is the essential modulator of neovascularization and diminished levels of VEGF-A have been associated with the impaired collateral vessel formation in the myocardial tissue of diabetic patients [18,19]. Using immunohistochemistry, we analyzed VEGF-A expression in histological sections of *Wt* and *Hif1a*^*+/-*^ hearts from diabetic and non-diabetic mice (Figure 6A). The anti-VEGF-A staining was found to be limited to the wall of coronary vessels in all groups. The relative quantification of VEGF-A expression in the wall of coronary vessels showed decreased protein levels by 50% in non-diabetic *Hif1a*^*+/-*^ compared to *Wt*, corresponding to the haploinsufficiency of *Hif1a* (Figure 6B). Furthermore, the VEGF-A protein levels were significantly reduced in the coronary vessels of diabetic *Hif1a*^*+/-*^ and *Wt* compared to non-diabetic *Wt*, indicating microvascular changes in the diabetic heart. Overall, these data suggest that the partial deficiency of *Hif1a* alters molecular and cellular adaptations of cardiac tissue to diabetic conditions.

## Discussion

This study investigated the functional role of HIF1-pathways in cardiac responses to diabetic conditions, including changes in echocardiographic parameters, transcriptional profile modulations, and tissue remodeling. For the first time, we showed that the partial deficiency of *Hif1a* accelerated the early-phase pathological effects of diabetes on the heart. The echocardiographic parameters of the LV were significantly affected in diabetic *Hif1a*^*+/-*^ animals. Impaired LV function of diabetic *Hif1a*^*+/-*^ mutants was accompanied by molecular changes associated with cardiac remodeling.

We used the STZ model which has been proved to produce diabetes in animal models without systemic toxicity and is characterized by hyperglycemia (blood glucose levels > 13.9) and insulinopenia. Most studies using animal models with STZ-induced diabetes revealed a decreased myocardial contractility and increased stiffness, resulting in both systolic and diastolic dysfunction at later stages of the disease (reviewed in [30,31]). However, the onset of these changes, preceded by an altered gene expression, differs in individual studies and can be explained by differences in the severity of hyperglycemia, chronicity of diabetes, and experimental conditions. For example, both echocardiography and magnetic resonance imaging performed in the fourth week of diabetes in mice showed impaired indices of systolic and diastolic function [32]. Similarly, diabetic rats exhibited decreased maximal systolic elastance at this stage of diabetes, indicating impaired intrinsic myocardial contractility [33]. However, Hoit et al. [34] observed the first signs of contractile dysfunction in rats only 5 weeks after STZ injections and the overt systolic and diastolic dysfunction in 6 weeks. Consistent with this study, our experiments revealed only a minor decrease in relative LV wall thickness and unchanged fractional shortening in 5-week-diabetic *Wt* mice, indicating that heart function was still preserved at this stage. However, the harmful effects of diabetes were clearly more pronounced in *Hif1a*^*+/-*^ mice as illustrated by the significantly decreased FS. It suggests that *Hif1a*^*+/-*^ deficiency promotes the development of systolic dysfunction in the diabetes-exposed heart. The LV dysfunction in *Hif1a*^*+/-*^ mice was associated with expressional changes connected with cardiac remodeling. Our observations are in line with the increasing evidence that the HIF1-regulated pathways are compromised in the diabetic heart [15,17,18].

Our molecular analysis showed increased levels of *Cxadr*, *Il6st, Pdgfra,* and *Slc2a1* in the LV of both *Wt* and *Hif1a*^*+/-*^ diabetic hearts which corresponds to the onset of pathological processes associated with cardiac remodeling in diabetic cardiomyopathy. The overexpression of *Cxadr*, an adhesion molecule found at the intercalated disc and gap junctions of cardiomyocytes, produces cardiomyopathy in transgenic mice [35]. The transmembrane signal transduction protein gp130, encoded by *Il6st*, is a common receptor for the interleukin 6 family, which contributes to inflammatory processes, cardiac fibrosis, and possibly to the development of type 1 and type 2 diabetes [36]. The activation of PDGFR-α induces collagen deposition, fibrosis, and inflammatory responses in an infarcted myocardium [37]. Observed increased levels of *Slc2a1*, the insulin-independent glucose transporter, indicate an adaptation of the myocardium to the diabetic environment for better glucose uptake and utilization [38].

In our study, the combinatory effect of the *Hif1a*^*+/-*^ genotype and diabetes was detected in the expression of *Gata2*, *Ctss,* and *Tfgbr1*. The transcriptional factor GATA2 cooperates with HIF1-α and complements HIF-1 transcriptional regulation of pro-inflammatory genes in endothelial cells [39,40]. Thus, the increase of *Gata2* mRNA in the diabetic *Hif1a*^*+/-*^ heart may indicate a compensation of HIF-1α activity. Increased levels of *Ctss* positively correlate with extracellular matrix remodeling in the diabetic *Hif1a*^*+/-*^ heart because CTSS protease is involved in matrix degradation and collagen deposition [41]. Although the important regulatory role of HIF-1α in inflammation has been established [42], a cross-talk between CTSS and HIF-1 has not yet been observed. We showed an increased expression of *Tgfbr1* mRNA in the LV of the *Hif1a*^*+/-*^ diabetic hearts, suggesting the activation of TGF-β signaling, which is associated with maladaptive changes in the composition of the extracellular matrix and fibrosis [43]. A cross-talk between TGF-β and HIF-1 pathways has been shown in the transcriptional regulation of *Vefga,* and *Col1* genes [44,45].

In our study, the molecular changes associated with alterations of structural molecules and with the composition of the extracellular matrix were also shown in the protein levels. We detected a reduction in the gap-junctional phosphorylated form of Cx43 in the LV of the *Hif1a*^*+/-*^ diabetic heart, which has been associated with diabetes-induced structural remodeling and impaired ventricular contractions [29]. We also showed increased protein levels of Col1 in *Hif1a*^*+/-*^ diabetic hearts compared to other groups, indicating modifications of the extracellular matrix and the onset of fibrosis. However, our immunohistological analysis revealed that the substantial cellular effects of hyperglycemia, including myocyte hypertrophy or fibrosis, were absent at this stage of diabetic cardiomyopathy. This phenotype reconciles with STZ-induced diabetes models characterized by the impaired LV function in the absence of significant structural changes in the early phase of diabetic cardiomyopathy [31].

Under normal conditions, apoptosis is a protective mechanism which eliminates old, useless, and damaged cells. Under diabetic conditions, increased apoptosis is associated with diabetes-related tissue damage and cardiac remodeling in diabetic hearts [46]. Surprisingly, we observed an increased number of apoptotic cells in the diabetes-exposed *Wt* hearts but not in the *Hif1a*^*+/-*^ hearts. The decreased sensitivity of *Hif1a*^*+/-*^ cardiac tissue to apoptosis-induction signals may be a consequence of the HIF-1α partial deficiency to induce apoptosis via p53, BNIP3, or/and caspase-3 pathways. However, additional studies are required to determine which signaling pathways mediate these effects in the diabetic *Hif1a*^*+/-*^ heart.

Diabetic microvascular defects, associated with the increased incidence of chronic wounds and decreased post-ischemic vascularization, have been accompanied by a significant reduction of VEGF-A, a key HIF-1 target gene product [18,19,47]. Decreased levels of VEGF-A mRNA have been detected in the ventricles of diabetic patients when compared to controls [19]. The observed reduction of cardiac VEGF-A levels correlated with pathologically altered responses of diabetic patients to myocardial ischemia. In our study, we demonstrated the significantly decreased expression of *Vegfa* mRNA in diabetic *Hif1a*^*+/-*^ compared to diabetic *Wt* mice. Both transcription and RNA stability can be enhanced by HIF-1α in response to normal as well as pathological conditions [48]. We observed discrepancies in the amplitude of mRNA and protein levels in *Hif1a*^*+/-*^ and *Wt* diabetic hearts. Although we are unable to explain these discrepancies, they are likely caused by the specific regulation of VEGF-A at post-transcription, translation, and post-translation levels [48]. Our model provides the first evidence that HIF-1α regulates *Vegfa* expression in the diabetic heart. The decreased levels of *Vegfa* in the *Hif1a*^*+/-*^ diabetic heart correlate with LV dysfunction and myocardial remodeling.

Our results are indirectly supported by a study showing that the overexpression of *Hif1a* gene under the control of the myosin heavy chain promoter normalizes VEGF-A levels and inhibits fibrosis in hearts exposed to diabetes [22]. Unfortunately, Xue et al. have not evaluated the echocardiographic functional parameters of the mutant heart to provide a more complex analysis. The protective role of HIF-1α in acute cardiac ischemia is well known [13,49]. However, the constitutive expression of HIF-1α and chronic long-term activation of HIF-1α pathways over time induce cardiomyopathy in transgenic mice with HIF-1α cardiac-specific overexpression [49]. Thus, a strict regulation of HIF-1α and its associated adaptive pathways is necessary for the long-term preservation of heart function.

In our study, under normoglycemic conditions, we showed decreased HIF-1α protein levels in *Hif1a*^*+/-*^ compared to *Wt* hearts, reflecting *Hif1a* haploinsufficiency (Figure 4). The reduction of HIF-1α levels in nuclear fractions from *Hif1a*^*+/-*^ tissues is consistent with other reports [10,23,24]. Although HIF-α levels are decreased in *Hif1a*^*+/-*^ mice, these mice are indistinguishable from their *Wt* littermates but have impaired responses to hypoxia and ischemia [11,23,24]. Accordingly, we observed the same phenotype in both *Wt* and *Hif1a*^*+/-*^ mice under normoglycemic conditions, including echocardiographic, geometrical, and functional parameters. However, under STZ-induced diabetes, *Hif1a*^*+/-*^ mice exhibited faster deterioration of cardiac functional parameters associated with diabetic cardiomyopathy compared to diabetic *Wt* mice. Unexpectedly, HIF-1α protein levels were increased by 2.6-fold in diabetic hearts of *Hif1a*^*+/-*^ mutants compared to diabetic *Wt*, which may indicate a possible compensation for heterozygosity for the *Hif1a* knockout allele by changes in the rate of synthesis or degradation of HIF-1α mRNA or protein. However, based on our VEGF-A expression data, the HIF-1a functional activity is affected by the combination of *Hif1a* haploinsufficiency and diabetes. This is in line with other reports showing that diabetes-reduced VEGF-A expression is the result of decreased HIF-1α functional activity but not HIF-1α stabilization [15,18]. Furthermore, our results showing decreased VEGF-A and increased TGF-β signaling coincide with other reports investigating *Hif1a* gene deletion mutants [24,45].

The most important limitation of our study lies in the global nature of the *Hif1a* deletion. We are unable to determine which cell type or which combinations of cell types are contributing to the increased susceptibility of *Hif1a*^*+/-*^ mice to diabetic cardiomyopathy. At the same time, the global deletion of *Hif1a* may affect other tissues and it may indirectly escalate pathological functional and structural changes in the heart of *Hif1a*^*+/-*^ mutants. For example, this may include the neuronal effect of HIF-1α, which may contribute to cardiac dysfunction [13]. Still, our results represent new information, which may have important implications for understanding the mechanisms behind the functional and structural remodeling of the myocardium in response to diabetes.

## Conclusions

According to the data obtained with our mouse model, the loss of *Hif1a* functional allele contributes to the development of diabetic cardiomyopathy. The partial deficiency of *Hif1a* accelerates the progression of diabetic cardiomyopathy by significantly decreasing LV fractional shortening*.* This functional impairment has been accompanied by changes in the LV transcriptional profile, including *Vegfa*, and cardiac remodeling. Our results highlight a critical link between diabetes, HIF-1α regulation, and cardiovascular dysfunction. Furthermore, clinical studies have demonstrated that polymorphisms at the HIF1A locus influence the development of ischemic heart disease and have been associated with type 2 diabetes [50,51]. The results presented in this study further suggest that genetic variation at the HIF1A locus may also influence the increased risk for diabetic cardiomyopathy.

## Abbreviations

HIF-1α: Hypoxia-inducible factor 1α; Slc2a1: Glucose transporter 1; Vegfa: Vascular endothelial growth factor A; Flt1: Vegf receptor-1; Igf2: Insulin-like growth factor 2; Ldha: Lactate dehydrogenase A; Bnip3l: BCL2/adenovirus E1B interacting protein 3-like; Cxadr: Coxsackie virus and adenovirus receptor; Il6st: Interleukin 6 signal transducer; Tgfbr1: Transforming growth factor beta receptor I; Itgav: Integrin alpha V; Pdgfra: Platelet derived growth factor receptor alpha; Gata2: GATA binding protein 2; Ctss: Cathepsin S; Col1: Collagen 1; Cx43: Connexin 43.

## Competing interests

The authors declare that they have no competing interests.

## Authors’ contributions

RB designed and performed qPCR experiments and histological analysis. FK designed and performed echocardiographic evaluations. DS designed and performed morphological and morphometric evaluations. LS prepared animals and processed tissues for morphological exams. FP and JN assisted in mice treatment and performed echocardiographic examinations. GP designed the study, analysed data, and wrote the paper. All authors read and approved the final manuscript.

## Pre-publication history

The pre-publication history for this paper can be accessed here:

http://www.biomedcentral.com/1472-6823/14/11/prepub

## Supplementary Material

Additional file 1 Primer sequences used for quantitative real-time polymerase chain reaction. Click here for file
