# Supplementary material for: Partial deficiency of HIF-1α stimulates pathological cardiac changes in streptozotocin-induced diabetic mice
Source: BMC Endocr Disord. 2014 Feb 6;14:11. doi: 10.1186/1472-6823-14-11 (PMC3922431; doi:10.1186/1472-6823-14-11)
Supplement: Additional file 1 — Primer sequences used for quantitative real-time polymerase chain reaction. [file 1472-6823-14-11-S1.docx]

Additional file 1: Table S1. Primer sequences used for quantitative real-time polymerase chain reaction.

| **Gene symbol** | **RefSeq ID** | **Forward primer sequence** | **Reverse primer sequence** |
| --- | --- | --- | --- |
| *Hprt1* | NM_013556 | 5'-GCTTGCTGGTGAAAAGGACCTCTCGAAG-3' | 5'-CCCTGAAGTACTCATTATAGTCAAGGGCAT-3' |
| *Slc2a1* | NM_011400 | 5'-GGGCATGTGCTTCCAGTATGT-3' | 5'-ACGAGGAGCACCGTGAAGAT-3' |
| *Vegfa* | NM_001025250 | 5'-ACTGGACCCTGGCTTTACTG-3' | 5'-TGGGACTTCTGCTCTCCTTC-3' |
| *Flt1* | NM_010228.3 | 5'-GAGGAGGATGAGGGTGTCTATAGGT-3' | 5'-GTGATCAGCTCCAGGTTTGACTT-3' |
| *Igf2* | NM_001122736 | 5'-CGCGGCTTCTACTTCAGC-3' | 5'-GGGGTGGCACAGTATGTCTC-3' |
| *Ldha* | NM_001136069 | 5'-GCACTGACGCAGACAAGG -3' | 5'-TGATCACCTCGTAGGCACTG-3' |
| *Bnip3I* | NM_009761.3 | 5'-CCTCGTCTTCCATCCACAAT-3' | 5'-TTCTTGTGGTGAAGGGCTGT-3' |
| *Cxadr* | NM_001025192 | 5'-CCTGGGGTTGCAAATAAGAA-3' | 5'-GATCCATCCACGAAGCATCT-3' |
| *Il6st* | NM_010560.3 | 5'-GTTCCTGATCCTTCCAAGAGTCAT-3' | 5'-CACAACGCTTACATCAGTGAAATTG-3' |
| *Tgfbr1* | NM_009370.2 | 5'-CGCGCTGACATCTATGCAAT-3' | 5'-AGGTACAAGATCATAATAAGGCAACTGA-3' |
| *Itgav* | NM_008402.2 | 5'-TTCGCCGTGGACTTCTTC-3' | 5'-CTGGGTCGTGTTCGCTTT-3' |
| *Pdgfra1* | NM_011058 | 5'-GTCCCCATGCTTGAAAGGAA-3' | 5'-CATCGTCCGAAAGGAGGTTTT-3' |
| *Gata2* | NM_008090 | 5'-CCCAAGCTTCGATTCTGTGT -3' | 5'-TTGACTCAGCACAATCGTCTC-3' |
| *Ctss* | NM_021281.2 | 5'-CGCCAGCCATTCCTCCTT-3' | 5'-ATGATTCACATTGCCCGTACAG-3' |

Primers were designed using Primer3 software

Abbreviations: *Hprt1*, hypoxanthine guanine phosphoribosyl transferase; *Slc2a1*, solute carrier family 2, member 1; *Vegfa*, vascular endothelial growth factor A; *Flt1*,FMS-like tyrosine kinase 1; *Igf2*, insulin-like growth factor 2; *Ldha*, lactate dehydrogenase A; *Bnip3I*, BCL2/adenovirus E1B interacting protein 3-like; *Cxadr*, coxsackie virus and adenovirus receptor; Il6st, interleukin 6 signal transducer; *Tgfbr1*, transforming growth factor β receptor I; *Itgav*, integrin alpha V; *Pdgfra1*, platelet-derived growth factor α receptor; *Gata2*, GATA binding protein 2; *Ctss*, cathepsin S.
